# HPLC-DAD-ESI/MS Identification and Quantification of Phenolic Compounds in *Ilex paraguariensis* Beverages and On-Line Evaluation of Individual Antioxidant Activity

**DOI:** 10.3390/molecules18043859

**Published:** 2013-03-28

**Authors:** Renato G. Peres, Fernando G. Tonin, Marina F. M. Tavares, Delia B. Rodriguez-Amaya

**Affiliations:** 1 Department of Food Science, Faculty of Food Engineering, University of Campinas-UNICAMP, P.O. Box 6121, 13083-862 Campinas, SP, Brazil; 2 Department of Biosystems Engineering, Faculty of Animal Science and Food Engineering, University of São Paulo, 13635-900 Pirassununga, SP, Brazil; 3 Institute of Chemistry, University of São Paulo, P.O. Box 26077, 05513-970 São Paulo, SP, Brazil

**Keywords:** beverage, *Ilex paraguarienses*, phenolic compounds, HPLC-DAD-ESI-MS, antioxidant capacity

## Abstract

“Chimarrão” and “tererê” are maté (dried, toasted and milled *Ilex paraguariensis* leaves and stemlets) beverages widely consumed in South America. This paper describes the application of HPLC-DAD-ESI/MS method for the identification and quantification of caffeoylquinic acids (CQA), flavonol glycosides and purine alkaloids in these beverages. The beverage samples were prepared from commercial lots of maté from Southern Brazil. The caffeoylquinic acids, 4,5-diCQA, 3-CQA, 5-CQA, and 4-CQA were the major compounds, having 238–289, 153–242, 183–263, and 123–188 μg/mL, respectively, for chimarrão and 206–265, 122–218, 164–209, 103–169 μg/mL, respectively, for tererê. Caffeine also had high amounts while glycosides of quercetin and kaempferol were found at much lower levels. The individual antioxidant activity was also determined by an on-line system that measured their ABTS^•+^ radical scavenging activity, showing that the antioxidant capacity was not proportional to the concentrations of the phenolic compounds. 3-CQA, quercetina-3-*O*-ramnosylglucoside, and quercetina-3-*O*-glucoside were the major contributors to the antioxidant capacity, although the quercetin glycosides had concentrations less than 10 times that of 3-CQA.

## 1. Introduction

Maté or yerba maté refers to the dried, roasted and ground leaves and stemlets of *Ilex paraguarienses* St. Hilaire, used to prepare tea-like beverages widely consumed in South America. Typical beverages from maté consumed in Brazil are “chimarrão” (beverage prepared with hot water) and “tererê” (beverage prepared with cold water).

Some therapeutic properties have been claimed for mate infusions, such as antirheumatic, hypocholesterolemic, anti-thrombotic, anti-inflammatory, anti-obesity, anti-aging, hepatoprotective and diuretic effects [1,2,3,4]. Some of these properties have been attributed to the high content of phenolic compounds, especially caffeoyl derivatives [5,6,7,8,9].

High performance liquid chromatography with diode array and mass detectors (HPLC-DAD-MS) has been used to identify the phenolic compounds of *Ilex paraguariensis*. Using an atmospheric pressure chemical ionization (APCI) interface in the negative-ion mode and collision-induced dissociation (CID) of precursor ions, along with UV-diode array detection, Carini *et al*. [10] analyzed mate from Argentina and identified 10 constituents: three naturally occurring caffeoylquinic acid (neo-chlorogenic, chlorogenic, and crypto-chlorogenic acids), three isomeric dicaffeoyl quinic acids, rutin (quercetin-3-rutinoside), a diglycosyl derivative of luteolin, and two caffeoyl-glucoside isomers. Quantification was not carried out, however. Also analyzing Argentinian maté by the UV and MS (ESI) spectra, 28 compounds were identified by Bravo *et al*. [11], including seven of those reported by Carini *et al*. [10]. Percentages of the polyphenol groups were presented. Using direct infusion electrospray insertion mass spectrometry (ESI-MS), the main phenolic compounds identified in Brazilian green yerba maté aqueous and ethanolic extracts were: caffeic acid, quinic acid, caffeoyl glucose, caffeoylquinic acid, feruloylquinic acid, dicaffeoylquinic acid and rutin [12]. After the roasting process two new compounds were formed: caffeoylshikimic acid and dicaffeoylshikimic acid. Quantitative data was given in terms of the total phenolic content.

Some quantification of the phenolic compounds in *Ilex paraguariensis* were reported in other articles [6,8,9], confirming the predominance of chlorogenic acid derivatives, but the data were expressed as total contents or relative percentages. In order to be truly useful for Food Science, Nutrition and Human Health purposes, the concentrations of individual compounds should be determined.

The antioxidant properties of maté has also been studied in chemical and biological systems [5,7,10,11,12,13,14,15,16,17,18], including studies with humans. Water extracts of maté were evaluated with healthy volunteers and the results suggested that *Ilex paraguariensis* antioxidants reached the plasma, increased the aqueous phase antioxidant capacity of the plasma and inhibited copper-induced autoxidation of LDL [13]. Maté tea supplementation was also shown to lower plasma lipid peroxidation and to increase the level of leukocyte antioxidant enzyme gene expression in healthy nonsmoking women [14].

Maté has been reported to have comparable or superior antioxidant activity as green tea [19,20]. Most studies measured effects against free radicals in general, but Leonard *et al*. [21] showed potent antioxidant effects specifically against hydroxyl and superoxide radicals in both chemical and cell systems, as well as DNA-protective properties.

Numerous papers assessing the antioxidant activity of extracts of foods and beverages have been published. The values obtained, however, usually represent the total antioxidant capacity of the food. Although often correlated or attributed to some food constituent groups (e.g., polyphenols), these are presumed rather than direct measurements and the contribution of individual compounds is not known. In 2001, on-line HPLC methods for the evaluation of the individual antioxidant activity of food constituents were introduced [22,23]. The HPLC-separated analytes reacted postcolumn with preformed radical cation ABTS^•+^ [22], radical DPPH^•^ or inhibit luminol chemilumiscence (CL) [23]. In the on-line ABTS^+^ method, the eluate reacted with a stabilized solution of ABTS^•+^ radical and the absorbance is monitored at high wavelengths such as 720 nm or 600 nm. The solution with the radical has a deep blue color and if a quenching reaction occurred, there would be a loss of color and a negative peak will be registered. This method was applied to green and black tea samples, using HPLC-MS for identification of the phenolic compounds [24]. The application of the technique of coupling HPLC to on-line, post-column assays and parallel chemical analysis to identify bioactive compounds from complex mixtures was reviewed by Shi *et al.* [25].

The objectives of this work were to identify and determine the concentrations of the principal phenolic compounds in the Brazilian beverages chimarrão and tererê by HPLC-ESI/MS and HPLC-DAD and evaluate their individual contribution to the total antioxidant capacity through an on-line HPLC system.

## 2. Results and Discussion

### 2.1. Identification of the Phenolic Compounds and Purine Alkaloids

**Figure 1:**
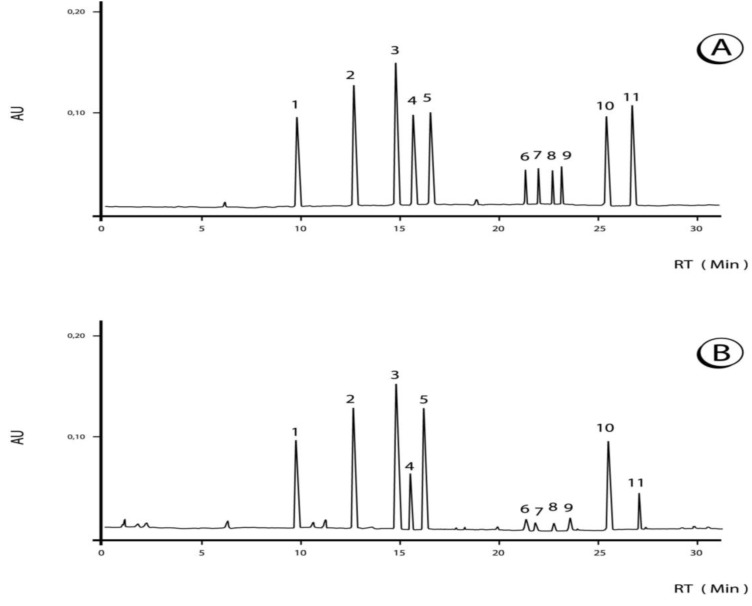
Chromatograms of (**A**) standards and (**B**) phenolic compounds of *Ilex paraguariensis* beverage “chimarrão” (sample S2). Peak identification: 1, caffeine; 2, 3-*O*-caffeoylquinic acid; 3, 5-*O*-caffeoylquinic acid; 4, teobromine; 5, 4-*O*-caffeoylquinic acid; 6, quercetin-3-rhamnosylglucoside; 7, quercetin-3-*O*-glucoside; 8, kaempferol-3-*O*-glucoside; 9, 3,5-dicaffeoylquinic acid; 10, 4,5-dicaffeoylquinic acid; 11, tricaffeoylquinic acid. Chromatographic conditions are described in the text. Detection was at 280 nm.

The typical chromatogram of the phenolics and purine alkaloids of *Ilex paraguariensis* beverages is shown in Figure 1. The chromatographic, HPLC-ESI/MS and UV data used for identification are presented in Table 1. The MS and UV spectral characteristics are comparable to those of Bravo *et al*. [11].

Peak 1 was identified as caffeine. It had maximum absorbance at 272 nm (with typical spectra of methylxanthine) and positively charged molecular ion [M+H]^+^ at *m/z* 195. It co-chromatographed with a caffeine standard.

Peaks 2, 3, and 5 had similar UV spectra (λ_max_ at 326 nm, shoulder at 296 nm), typical of chlorogenic acid. Their MS spectra exhibited [M+H]^+^ at *m/z* 355, indicative of isomers of chlorogenic acid (C_16_H_18_O_19_; M.W. 354), which had been reported as important and major constituents of maté [7]. Peak 3 co-chromatographed with the 5-CQA standard and peaks 2 and 5 with 3-CQA and 4-CQA standards prepared as described in the experimental section. The order of elution was the same as that of Bravo *et al.* [11] and Negishi *et al*. [26].

Peak 4 was identified as teobromine by the UV (λ_max_ at 272 nm) and MS (with [M+H]^+^ at *m/z* 181) spectra and co-chromatography with its standard.

Minor peaks 6, 7, and 8 were identified as flavonoids from the UV and MS spectral data. Peak 6 (λ_max_ at 256 and 354 nm, [M+H]^+^ at *m/z* 611) was identified as quercetin-3-rhamnosylglucoside (quercetin-3-rutinoside) known as rutin. Peak 7 (λ_max_ at 256 and 344 nm, [M+H]^+^ at *m/z* 465) was identified as quercetin-3-*O*-glucoside and peak 8 (λ_max_ at 266 and 342 nm, [M+H]^+^ at *m/z* 449) as kaempferol 3-*O*-glucoside. These compounds co-chromatographed with the respective standards.

**Table 1:** Identifying characteristics of the phenolic compounds and purine alkaloids of beverages from *Ilex paraguariensis*.

Peak No.	RT min	λ_max_ * nm	[M+H]^+^ *m/z*	Fragment ions *m/z*	Identification
1	13.9	**272**	195		Caffeine
2	16.8	**326**,296sh	355	193,181	3-*O*-Caffeoylquinic acid
3	19.8	**326**,296sh	355	193,181	5-*O*-Caffeoylquinic acid
4	19.8	**272**	181		Teobromine
5	20.1	**326**,296sh	355	193,181	4-*O*-Caffeoylquinic acid
6	24.2	256,**354**	611		Quercetin-3-rhamnosylglucoside
7	25.0	256,**344**	465	303	Quercetin-3-*O*-glucoside
8	25.6	266,**342**	449		Kaempferol-3-*O*-glucoside
9	27.4	**326**,296sh	517	355,193	3,5-Dicaffeoylquinic acid
10	27.8	**326**,296sh	517	355,193	4,5-Dicaffeoylquinic acid
11	28.5	**326**,296sh	679	517,181,163	Tricaffeoylquinic acid

* The wavelengths of maximum absorption are in bold; sh, shoulder.

Peaks 9 e 10 both had [M+H]^+^ at *m/z* 517, indicative of dicaffeoylquinic acid isomers, which were found in maté leaves by some authors [7,9]. The loss of one dehydrated caffeic acid molecule [M-caffeic acid-H_2_O]^+^, giving the fragment at 355 *m/z*, the elution profile similar to that reported in C_18_ columns by others authors [9,27], and the UV data (λ_max_ at 326 nm, shoulder at 296 nm) all agreed with the identification of peaks 9 and 10 as 3,5-dicaffeoylquinic and 4,5-dicaffeoylquinic acids. Additionally, peak 9 co-chromatographed with a standard of 3,5-dicaffeoylquinic acid.

Another major compound found in the maté beverages was peak 11 with UV spectra (λ_max_ at 326 nm, shoulder at 296 nm) and [M+H]^+^ at *m/z* 679 of a caffeoyl quinic derivative. The fragment at *m/z* 517 could be attributed to tricaffeoylquinic acid with a loss of a dehydrated molecule of caffeic acid that corresponded to the fragment at *m/z* 163. Tricaffeoylquinic acid was cited by Yoshimoto *et al*. [27] in sweet potato leaves and by Bravo *et al.* [11] in maté leaves, identified by HPLC/MS.

### 2.2. Quantification of the Phenolic Compounds and Purine Alkaloids

The method showed good performance. Correlation coefficients for the standard curves varied from 0.99983 to 0.99998. The limit of detection was 0.02 to 0.1 μg/mL and the limit of quantification was 0.1 to 0.5 μg/mL. Repeatibility for retention time and peak area was excellent, the coefficients of variation (CV%) for 10 consecutive injections ranging from 0.0019 to 0.008 and 0.02 to 0.03, respectively. Percent recovery varied from 97 ± 2 to 101 ± 4%.

Table 2 shows the concentrations of phenolic acids and purine alkaloids of the chimarrão and tererê beverages prepared from maté. The methylxanthine caffeine varied from 124 to 268 μg/mL for chimarrão and from 114 to 246 μg/mL for tererê, while teobromine varied from 64 to 126 μg/mL for chimarrão and 54 to 114 μg/mL for tererê. The substantial variation among the commercial samples analyzed indicated that there should be better quality control. Compositional variation is expected because of the influence of such factors as variety planted, soil composition, maturity at harvest, climate, post-harvest handling and processing/storage conditions. The present results agree with those of Clifford and Ramirez-Martinez [6], in which caffeine levels were approximately twice as much as those of teobromine. Bastos *et al*. [28] encountered slightly higher values (150–294 μg/mL) for caffeine in chimarrão prepared from *Ilex paraguariesis* from the state of Parana. The samples in the present work came from three states.

The ranges for 3-CQA, 5-CQA, and 4-CQA in the present work were 153–242, 183–263, and 123–188 μg/mL, respectively, for chimarrão and 122–218, 164–209, 102–169 μg/mL, respectively for tererê. Values in the literature are given as chlorogenic acid or 5-CQA, without distinguishing the three isomers.

Three flavonol glycosides (quercetin-3-*O*-rhamnosylglucoside, quercetin-3-*O*-glycoside and kaempferol-3-*O*-glycoside) were detected and quantified (Table 2). The first glycoside varied from 8 to 18 μg/mL in chimarrão and from 7 to 12 μg/mL in tererê. The ranges for the second glycoside were 6–13 μg/mL for chimarrão and 4–12 μg/mL for tererê, and for the third glycoside trace levels to 16 μg/mL for chimarrão and trace levels to 15 μg/mL for tererê. The flavonol concentrations presented in the literature are in terms of the aglycone, not the glycoside form. Comparison of quantification in the glycoside and aglycone forms is the subject of another paper.

**Table 2:** Concentrations (µg/mL) of phenolic compounds and purine alkaloids of beverages of *Ilex paraguariensis*.

Compound	S1		S2		S3		S4		S5		S6	
	Chimarrão	Tererê	Chimarrão	Tererê	Chimarrão	Tererê	Chimarrão	Tererê	Chimarrão	Tererê	Chimarrão	Tererê
Caffeine	146 ± 11	124 ± 9	265 ± 12	234 ± 10	190 ± 13	168 ± 9	124 ± 8	114 ± 9	268 ± 12	246 ± 10	197 ± 15	175 ± 11
3-*O*-Caffeoylquinic acid	183 ± 6	167 ± 4	153 ± 7	122 ± 5	193 ± 8	168 ± 8	166 ± 4	135 ± 7	189 ± 5	169 ± 6	242 ± 9	218 ± 4
5-*O*-Caffeoylquinic acid	263 ± 4	203 ± 5	212 ± 9	199 ± 6	183 ± 8	164 ± 10	213 ± 12	209 ± 7	219 ± 8	193 ± 6	210 ± 9	185 ± 6
Teobromine	72 ± 3	57 ± 3	125 ± 9	114 ± 8	102 ± 6	77 ± 5	64 ± 7	54 ± 4	126 ± 8	113 ± 1	90 ± 4	73 ± 3
4-*O*-Caffeoylquinic acid	153 ± 8	125 ± 4	188 ± 4	169 ± 9	139 ± 8	114 ± 12	153 ± 7	133 ± 1	130 ± 5	104 ± 3	123 ± 8	102 ± 6
Quercetin-3-rhamnosylglucoside	10 ± 1	9 ± 1	12 ± 2	11 ± 2	10 ± 1	8 ± 1	10 ± 1	8 ± 1	18 ± 1	12 ± 1	8 ± 1	7 ± 1
Quercetin-3-*O*-glucoside	12 ± 1	10 ± 1	13 ± 1	12 ± 1	13 ± 2	7 ± 1	8 ± 1	6 ± 1	10 ± 1	11 ± 1	6 ± 1	4 ± 1
Kaempferol-3-*O*-glucoside	9 ± 1	7 ± 1	10 ± 1	8 ± 1	tr	tr	tr	tr	12 ± 1	13 ± 1	16 ± 1	15 ± 1
3,5-Dicaffeoylquinic acid	112 ± 7	103 ± 8	138 ± 8	112 ± 8	167 ± 7	123 ± 3	145 ± 5	123 ± 6	156 ± 3	126 ± 4	165 ± 4	145 ± 5
4,5-Dicaffeoylquinic acid	238 ± 8	206 ± 7	278 ± 6	254 ± 7	289 ± 6	256 ± 8	267 ± 7	224 ± 8	279 ± 5	239 ± 6	287 ± 5	265 ± 8
Tricaffeoylquinic acid *	125 ± 9	102 ± 5	128 ± 7	106 ± 4	156 ± 6	134 ± 5	136 ± 4	108 ± 5	143 ± 6	139 ± 6	154 ± 4	143 ± 5
Total phenolics	1105	932	1132	993	1150	974	1098	946	1156	1016	1211	1084

S1 to S2 are beverages prepared from 6 different commercial sample lots of maté from 3 Brazilian states; n = 6, for each sample lot, 2 beverage samples were prepared, each of which was injected in triplicate. * Estimated from the 3,5-dicaffeoylquinic acid calibration curve. tr: trace levels.

For 3,5-dicaffeoylquinic acid, the ranges were 112–167 μg/mL for chimarrão and 103–145 μg/mL for tererê; the corresponding ranges for 4,5-dicaffeoylquinic acid were 238–289 and 206–265 μg/mL. Comparison could not be made with the literature because these acids were quantified in terms of the dried leaves [8]. Tricaffeoylquinic acid ranged from 125 to 156 μg/mL in chimarrão and 102 to 143 μg/mL in tererê. This acid has not been previously quantified. 

It is noteworthy that the concentrations of all the compounds quantified were lower in tererê than in chimarrão, which is coherent with the fact that the former was prepared with cold water and the latter with hot water, reflecting better extraction of the compounds studied in the latter beverage.

### 2.3. Antioxidant Activity of the Phenolic Compounds

**Figure 2:**
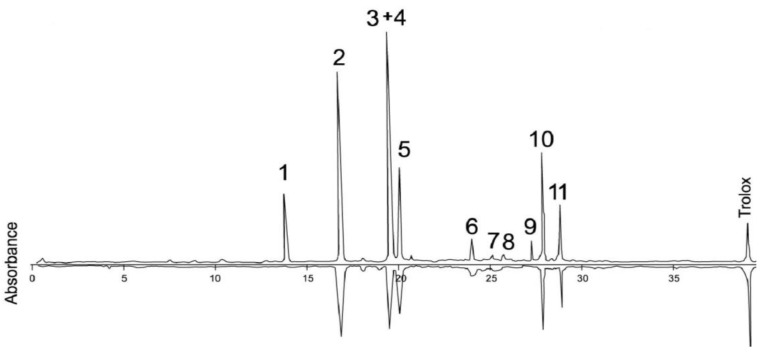
On-line HPLC ABTS^+^ analysis of the *Ilex paraguarienses* beverage chimarrão (sample S2). Positive Trace: PDA at 280 nm and Negative Trace: UV-VIS at 600 nm. Peak identification: 1, caffeine; 2, 3-*O*-caffeoylquinic acid; 3, 5-*O*-caffeoylquinic acid; 4, teobromine; 5, 4-*O*-caffeoylquinic acid; 6, quercetin-3-rhamnosylglucoside; 7, quercetin-3-*O*-glucoside; 8, kaempferol-3-*O*-glucoside; 9, 3,5-dicaffeoylquinic acid; 10, 4,5-dicaffeoylquinic acid; 11, tricaffeoylquinic acid. Chromatographic conditions are described in the text.

Figure 2 shows the HPLC profile of the beverage chimarrão (positive trace indicates the absorbance obtained with the DAD detector at 280 nm and the negative trace the absorbance obtained by the second detector at 600 nm). In the column used for the antioxidant activity, teobromine co-eluted with 5-CQA, giving the highest positive peak and a small negative peak because teobromine is not an antioxidant.

Table 3 displays the mean concentrations of the phenolic compounds and the TEAC value, clearly showing that the antioxidant capacity was not proportional to the analytes’ concentrations. 3-CQA, quercetin-3-*O*-glucoside, quercetin-3-rhamnosylglucoside had the highest TEAC value (1.0), although the quercetin glycosides had concentrations less than 10 times that of 3-CQA. Tri-CQA, kaempherol-3-*O*-glucoside, and 4-*O*-CQA had TEAC value of 0.9, although the CQAs had levels more than 10 times that of the kaempferol glycoside. The major phenolic compounds of the mate infusions, 4,5-diCQA and 5-CQA, had lower TEAC values. Similar results were obtained with the beverage tererê (data not shown).

**Table 3:** Individual antioxidant capacity of the phenolic compounds of the beverage chimarrão of *Ilex paraguariensis.*

Compound	Mean Concentration (µg/mL)	TEAC (a_c_/a_T_)
3-*O*-Caffeoylquinic acid	188 ± 12	1.0 ± 0.1
5-*O*-Caffeoylquinic acid	217 ± 13	0.8 ± 0.1
4-*O*-Caffeoylquinic acid	148 ± 9	0.9 ± 0.1
Quercetin-3-rhamnosylglucoside	11 ± 1	1.0 ± 0.1
Quercetin-3-*O*-glucoside	10 ± 2	1.0 ± 0.1
Kaempferol-3-*O*-glucoside	12 ± 1	0.9 ± 0.1
3,5-Dicaffeoylquinic acid	147 ± 9	0.8 ± 0.1
4,5-Dicaffeoylquinic acid	273 ± 13	0.8 ± 0.1
Tricaffeoylquinic acid	140 ± 10	0.9 ± 0.1
Trolox	-	1.0

n = 6, for each sample lot, 2 beverage samples were prepared, each of which was injected in triplicate. TEAC, Trolox equivalent antioxidant capacity; a_c_, slope of compound calibration curve; a_T_, slope of Trolox calibration curve.

## 3. Experimental

### 3.1. Chemicals

Standards of the flavonoids quercetin-3-rhamnosylglucoside, quercetin-3-*O*-glucoside, kaempferol-3-*O*-glucoside and 3,5-dicaffeoylquinic acid were purchased from Extrasynthese (Genay, France); caffeine, teobromine and 5-*O*-cafeoylquinic acid were obtained from Sigma Chemical Company (St. Louis, MO, USA). HPLC-grade methanol and acetonitrile were purchased from Tedia Company (Fairfield, OH, USA). The 2,2'-azinobis-(3-ethylbenzothiazoline-6-sulfonic acid) was purchased from ICN Biomedicals Inc. (Aurora, OH, USA) and 6-hydroxy-2,5,7,8-tetramethylchroman-2-carboxylic acid (Trolox) and butyl-hydroxyanisol (BHA), from Aldrich (St. Louis, MO, USA). Membrane filters were purchased from Millipore (Bedford, MA, USA). Stock standard solutions were prepared at a concentration of 1 mg/mL in methanol and stored at −20 °C for a maximum of two months. Water was purified using a Milli-Q system from Millipore Corporation (Bedford, MA, USA).

### 3.2. Sample Preparation

The maté samples (S1 to S6) were purchased from supermarkets in São Paulo, Brazil, and were produced in the states of Paraná, Santa Catarina, and Rio Grande do Sul (two sample lots from each state). Two packages of each sample lot were ground to pass a 40-mesh screen and mixed.

Chimarrão was prepared in duplicate by adding 250 mL of deionized water at 70 °C to 5 g of the sample. The infusion was stirred for 3 min and then filtered through a 0.45 µm membrane filter (Millipore, Bedford, MA, USA). Tererê was prepared using the same procedure, but the water temperature was 5 °C.

An aliquot of chimarrão or tererê was diluted 20-fold with deionized water and injected in the HPLC system.

### 3.3. Preparative HPLC

3-Caffeoylquinic acid and 4-CQA have the same MS and UV spectra and are not commercially available as standards. So it was necessary to prepare [29] and isolate them by preparative HPLC [26]. For this, the isomers were separated and collected using a preparative Phenomenex C_18_ column, 15 μm, 250 × 21.2 mm (Torrance, CA, USA). The mobile phase and gradient system were those employed by Negishi *et al*. [26]. These authors identified the isomers by MS and NMR. The preparative HPLC procedure was performed on a Waters Prep System (Milford, MA, USA), comprising of a HPLC Prep pump model 600E, Rheodyne injector, dual absorbance detector model 2487, a preparative flow cell, and a fraction collector (WFC), connected to a recorder model BD40 (Kipp & Zonen, Delft, The Netherlands).

### 3.4. HPLC Analysis

The HPLC equipment used for the separation and quantification of the phenolic acids consisted of a Waters alliance model 2695 separation module with a photodiode array detector model 996, equipped with an X-Bridge C_18_ column, 5µm, 4.6 × 150 mm, a microbore detection cell and, for data collection and treatment, an Empower 2 software (Waters Corporation, Milford, MA, USA). The system was thermostated at 35 °C with a Waters temperature control module and the flow rate was 1.0 mL/min. A binary convex gradient was employed with the following solutions: H_2_O/formic acid (99:1 v/v) as phase A and acetonitrile/formic acid (99:1 v/v) as phase B. Starting with 95A/5B, the proportion was altered to 30A/70B in 5 min, then to 20A/80B in 15 min, and to 95A/5B in 30 min. The injection volume was 10 µL.

Quantification was done by external standardization, using the respective standards, at the wavelengths of maximum absorption of the compounds. However, tricaffeoylquinic acid, for which there is no commercially available standard, was estimated from the 3,5-dicaffeoylquinic acid curve. Each standard curve was constructed with 6 different concentrations, covering the expected concentrations of the samples, each concentration being injected in triplicate.

Linearity was evaluated by the injection of 6 standard solutions for each polyphenol at concentrations varying from 1.25 to 4.00 µg/mL. Limits of Detection (LOD) and Quantification (LOQ) were calculated as the concentrations that gave signal-to-noise ratios of 3 and 10, respectively. Peak retention time and area were evaluated by 10 consecutive injections of the standard solutions. For the recovery test, samples were spiked at three levels of the standard for each polyphenol, each level being evaluated in triplicate. Since the concentrations of the polyphenols in the samples varied widely, the levels of added standards varied from 5 to 300 µg/mL. Preliminary quantification was carried out to insure that the levels added bracketed those found in the samples.

### 3.5. HPLC-ESI/MS Analysis

The extracts were analyzed by a Micromass ZMD quadrupole mass spectrometer, with electrospray interface and MassLynx software version 3.5 (Micromass UK Ltd, Manchester, UK) for data acquisition. This instrument was coupled to a Waters separation module, model 2695, equipped with a photodiode detector model 996 and a microbore cell (Waters Corporation, Milford, MA, USA). The mass spectrometer parameters were set as follows: ionization mode, electrospray positive ion; capillary voltage, 4.0 kV; source block temperature, 140 °C; desolvation temperature, 400 °C; nebulizer nitrogen flow rate, 50 L/h; desolvation nitrogen gas flow, 568 L/h; LM resolution, 10; HM resolution, 12; ion energy, 1.0 V; cone voltage, 35 V; RF lens, 0.5 V; extractor lens, 5 V. Spectra were recorded by scanning the mass range from *m/z* 100 to 1000 with scan time of 1 s.

### 3.6. Preparation of the ABTS^•+^ Solution

ABTS (2 mmol/L) was dissolved in 10 mmol/L phosphate-buffered solution and adjusted to pH 7.4 with ammonium persulfate solution in ultrapure water (0.5 mmol/L final concentration) to produce the ABTS^•+^. To maximize the conversion of ABTS to ABTS^•+^, the mixture was prepared in an amber glass volumetric flask and stored in the dark at room temperature for 16 h under constant stirring. Fresh working solutions of the appropriate concentrations were prepared daily; the final dilution was 1:40 v/v in water.

### 3.7. On-line ABTS^•+^ Assay

**Figure 3:**
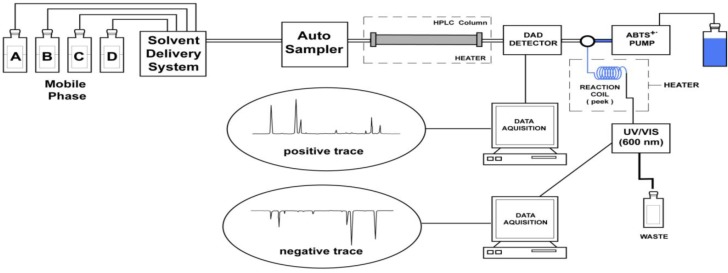
Set-up for the HPLC-ABTS^•+^ on-line system.

Determination of the antioxidant activity was based on the ABTS^•+^ assay of Koleva *et al*. [22]. The on-line set-up utilized in the present study is shown in Figure 3. The HPLC equipment consisted of a Shimadzu HPLC system pump model LC-10ADvp, system controller model SCL-10Avp, an autosampler Waters model 717Plus, DAD detector model SPD-M10Avp, controlled by a Class VP software v. 5.03. Separation was carried out on a Synergi Max-RP C_12_ column, 4 µm, 4.6 × 250 mm (Phenomenex, Torrance, CA, USA) and the flow rate was 1.0 mL/min. The HPLC column and the PEEK (polyether ether ketone) tubing were maintained at 35 °C, using a Waters column heater, model TCM/CHM (Waters Corporation, Milford, MA, USA). When the HPLC eluent from the DAD detector (recorded from 200 to 600 nm and monitored at 280 nm) arrived at a connection in “T” form (Altech, Deerfield, IL, USA) where the ABTS^•+^ was added, the compound reacted with the cation radical and decolorization was monitored by a second detector, a Waters UV/VIS tunable absorbance detector, model 486. The antioxidant on-line part of the system was controlled and monitored by Waters Empower 2 software. The ABTS^•+^ solution flow rate was 0.8 mL/min, delivered by a Waters 515 HPLC pump. After mixing through a 13.7 × 0.25 mm i.d. PEEK tubing (Upchurch Scientific Corporation, Oak Harbor, WA, USA) (used as reaction coil), the absorbance was measured by a Waters tunable absorbance detector, model 486, set at 600 nm.

To calculate the antioxidant activity, Trolox was injected with the extracts at three concentrations (ranging from 5.0 to 50.0 µg/mL) to construct the calibration curves. Initially curves consisting of 6 points, each point in triplicate, were utilized. Because the curves had to be constructed daily, and the daily volume of samples was large, 3-point curves bracketing the concentrations of the samples were subsequently used. In a comparison of the two curves, the six-point curve had R = 0.99987 and the three-point curve had R = 0.99985. The Trolox equivalent antioxidant capacity (TEAC) was calculated by dividing the slope of the compound’s curve by the slope of the Trolox curve [22].

## 4. Conclusions

The major phenolic compounds of *Ilex paraguiariensis* beverages are the caffeoylquinic acids 4,5-diCQA, 3-CQA, 5-CQA, and 4-CQA. However, along with 3-CQA, quercetin-3-*O*-ramnosylglucoside, and quercetin-3-*O*-glucoside appear to be the principal contributors to the antioxidant capacity, although their concentrations are 10 times less than that of 3-CQA.
